# Curcumin enhances elvitegravir concentration and alleviates oxidative stress and inflammatory response

**DOI:** 10.1038/s41598-023-47226-1

**Published:** 2023-11-14

**Authors:** Sandip Godse, Lina Zhou, Namita Sinha, Sunitha Kodidela, Asit Kumar, Udai P. Singh, Santosh Kumar

**Affiliations:** Department of Pharmaceutical Sciences, College of Pharmacy, The University of Tennessee Health Science Center, Memphis, TN 38163 USA

**Keywords:** Biochemistry, Drug discovery, Immunology, Microbiology, Diseases

## Abstract

In this study, we investigated the potential of using curcumin (CUR) as an adjuvant to enhance the delivery of antiretroviral drug elvitegravir (EVG) across the BBB, and alleviate oxidative stress and inflammatory response, which are the major hallmark of HIV neuropathogenesis. In a mouse model, we compared the biodistribution of EVG alone and in combination with CUR using intraperitoneal (IP) and intranasal (IN) routes. IN administration showed a significantly higher accumulation of EVG in the brain, while both IP and IN routes led to increased EVG levels in the lungs and liver. The addition of CUR further enhanced EVG brain delivery, especially when administered via the IN route. The expression of neural marker proteins, synaptophysin, L1CAM, NeuN, and GFAP was not significantly altered by EVG or CUR alone or their combination, indicating preserved neural homeostasis. After establishing improved brain concentration and safety of CUR-adjuvanted EVG in mice in acute treatment, we studied the effect of this treatment in HIV-infected U1 macrophages. In U1 macrophages, we also observed that the addition of CUR enhanced the intracellular concentration of EVG. The total area under the curve (AUC_tot_) for EVG was significantly higher in the presence of CUR. We also evaluated the effects of CUR on oxidative stress and antioxidant capacity in EVG-treated U1 macrophages. CUR reduced oxidative stress, as evidenced by decreased reactive oxygen species (ROS) levels and elevated antioxidant enzyme expression. Furthermore, the combination of CUR and EVG exhibited a significant reduction in proinflammatory cytokines (TNFα, IL-1β, IL-18) and chemokines (RANTES, MCP-1) in U1 macrophages. Additionally, western blot analysis confirmed the decreased expression of IL-1β and TNF-α in EVG + CUR-treated cells. These findings suggest the potential of CUR to enhance EVG permeability to the brain and subsequent efficacy of EVG, including HIV neuropathogenesis.

## Introduction

While antiretroviral therapy (ART) has been successful in controlling HIV replication and improving overall health outcomes, it fails to adequately address the neuronal complications associated with the virus^1^. One of the key factors contributing to this limitation is the existence of viral reservoirs within the central nervous system (CNS), particularly in perivascular macrophages and microglia, which serve as hiding places for latent HIV^2^. Despite effective systemic viral suppression, the CNS can act as a reservoir for persistent viral replication,, leading to chronic inflammation, release of cytokines, and cellular damage^3^. These factors collectively contribute to the development of HIV-associated neurocognitive disorder (HAND)^3^. The prevalence of HAND continues to rise, indicating the ongoing progression of neuronal damage in individuals living with HIV, regardless of virological and immunological indicators^1^. Viral RNA has even been detected in the brains of individuals with fully suppressed plasma viral load, primarily due to the limited ability of current ART drugs to effectively penetrate the BBB and suppress viral replication within the CNS^4^. This incomplete suppression of viral activity can result in neuronal injury or dysfunction, as prolonged exposure to inflammatory responses and neurotoxic viral proteins perpetuates neuronal damage.

The eradication of latent HIV reservoirs from the CNS and the management of neuronal complications present significant challenges in the treatment of HAND. Poor penetration of ART drugs into the CNS, viral replication-induced neuronal damage, and potential neurotoxicity associated with ART drugs contribute to suboptimal viral suppression and inadequate neuronal protection^5, 6^. Additionally, the selective nature of the BBB, efflux transporters, e.g., P-glycoprotein (P-gp) in BBB and myeloid cells, and the presence of metabolic enzymes, e.g., cytochrome P4503A4 in myeloid cells further limit the concentrations of ART drugs in the CNS^5, 7–9^. To address these challenges, recent advancements in the field have explored novel strategies such as nanoparticulate ART, ART nanosuspensions, and monocyte/macrophage targeted ART approaches^10–12^. These approaches have shown promising results in humanized HIV-infected mice, suggesting that enhancing CNS ART delivery can have neuroprotective effects. Re-formulated ART preparations with longer-lasting effective drug release are also being considered for new human clinical trials, specifically targeting HIV-associated neurocognitive impairment^13^. Interestingly, despite the initiation of combination ART (cART), the overall prevalence of HAND remains unchanged^3^. This suggests that viral load alone is not the crucial driver for the development of HAND. Emerging evidence suggests that self-fueling inflammatory processes within the CNS contribute to neurodegeneration^14^. Thus, targeting these inflammatory processes, independent of viral load, with immunomodulatory drugs in addition to cART, presents an intriguing approach to reduce chronic inflammation and maintain cognitive function in HAND patients.

In recent years, there has been a growing interest in exploring natural compounds or nutraceuticals with anti-HIV activity to enhance therapeutic outcomes in neuroHIV and HAND^15–17^. While studies investigating natural compounds as potential anti-inflammatory agents for HAND treatment are limited, CUR has emerged as a promising candidate. CUR, the primary curcuminoid derived from the rhizome of Curcuma longa, possesses diverse biological properties, including anti-inflammatory, antioxidant, and antimicrobial effects^18, 19^. It has demonstrated anti-viral activity against a wide range of viruses, including HIV, and has shown neuroprotective effects in the context of neurodegenerative diseases^20, 21^. CUR's ability to reduce neuroinflammation, protect against oxidative damage, and inhibit the formation of amyloid fibrils associated with neurocognitive impairments makes it a promising adjuvant. In our study, we selected EVG as our therapeutic agent. EVG is an FDA-approved integrase strand transfer inhibitor and a key component of one of the initial ART regimens for newly diagnosed HIV patients in the USA^22^. In addition, EVG has better safety profile than other antiretrovirals^23^. However, its ability to cross the blood–brain barrier is limited, with a low CSF-plasma drug concentration ratio of approximately 0.3% in HIV-infected individuals^24^. Our hypothesis is that CUR, especially using IN delivery, enhances the transmigration of the ART drug EVG in macrophages and in rodent brain. We also hypothesize that CUR alone and with EVG combination result in reduced oxidative stress and inflammation, the major hallmarks of HIV neuropathogenesis and HAND.

## Results

### Effect of CUR on biodistribution of EVG at high dose via IP and IN route in mice

**Figure 1:**
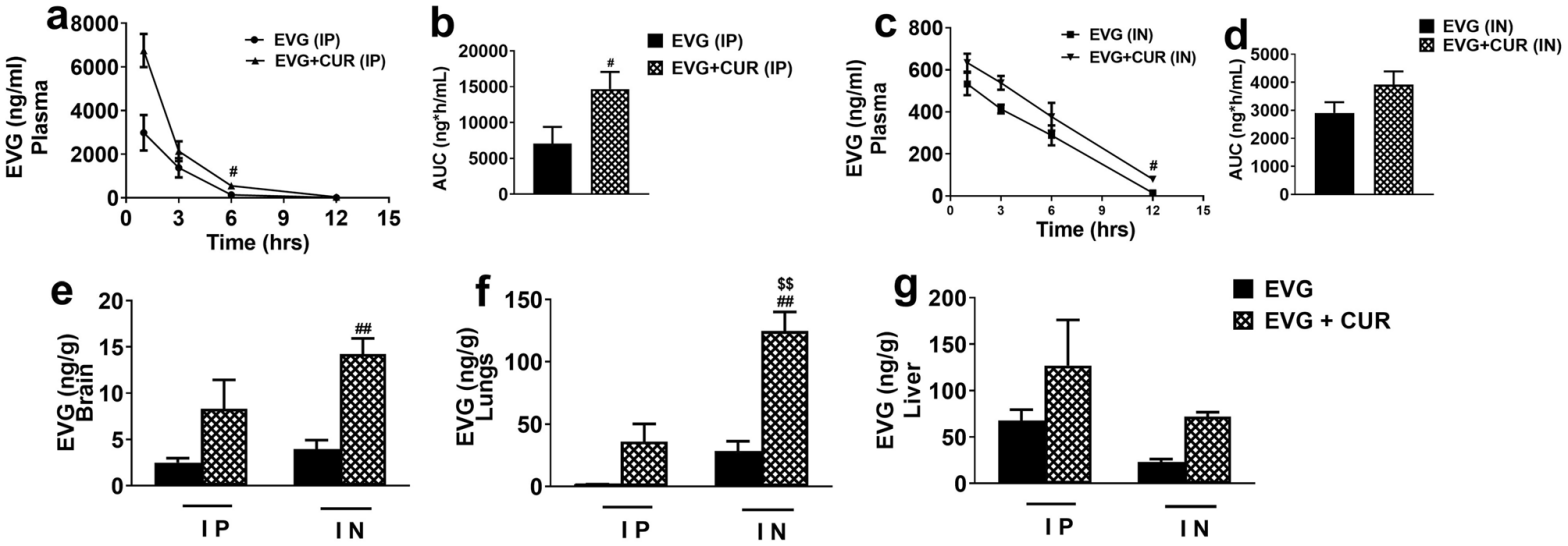
Biodistribution of EVG (25 mg/kg) in presence of CUR (20 mg/kg) administered via IP and IN in Balb/c mice. The concentrations of EVG were measured in plasma (**a**–**d**) at 1, 3, 6, and 12 h and in the brain (**e**), lungs (**f**), and liver (**g**) at 12 h. Concentrations are expressed as ng/g in tissues and ng/ml in plasma. Statistical analysis was performed using ANOVA (multiple comparisons) and t-test (two groups). Results are expressed as means ± S.E.M (n = 6). #, and ## represent *p* ≤ 0.05, and *p* ≤ 0.01 respectively, when compared to EVG. $$ represent *p* ≤ 0.01 when compared to EVG + CUR (IP).

The primary aim of this study is to compare the in vivo biodistribution of EVG, both as a standalone drug and in combination with CUR, through the utilization of two different administration routes: IP and IN. In the first phase of the experiment, we investigated the distribution of EVG (25 mg/kg) in combination with CUR (20 mg/kg) following IP administration in the brain, liver, lungs, and plasma for 12 h. In this study we only measured the concentration of EVG, not its metabolites. The results demonstrated that the addition of CUR significantly increased the accumulation of EVG in the plasma for IP administration (Fig. 1a and b, #*p* ≤ 0.05). There was a pattern of increase in EVG concentrations by CUR in the plasma for IN administration, however, they were not statistically significant (Fig. 1c and d). Although not statistically significant, a similar trend of relatively high EVG concentrations in combination therapy was observed in the brain (Fig. 1e), lungs (Fig. 1f), and liver (Fig. 1g) when compared to EVG (IP) treatment alone. In the subsequent phase of the experiment, we evaluated the impact of IN administration on the biodistribution of EVG in the presence of CUR. Our findings indicated a significant increase in EVG accumulation in the brain (Fig. 1e) when compared to EVG (IN) only group (###*p* ≤ 0.001) and EVG (IP) only group ($$$*p* ≤ 0.001). EVG concentration in lungs (Fig. 1f, ##*p* ≤ 0.01, $$*p* ≤ 0.01) also appeared to be increased in EVG + CUR (IN) group. A similar trend, with increased EVG concentrations in combination therapy, was also observed in plasma and liver, although the effect was not statistically significant. These results highlight the potential of CUR to enhance the brain delivery of EVG, especially for IN administration.

### Effect of CUR on biodistribution of EVG at low dose via IP and IN route in mice

**Figure 2:**
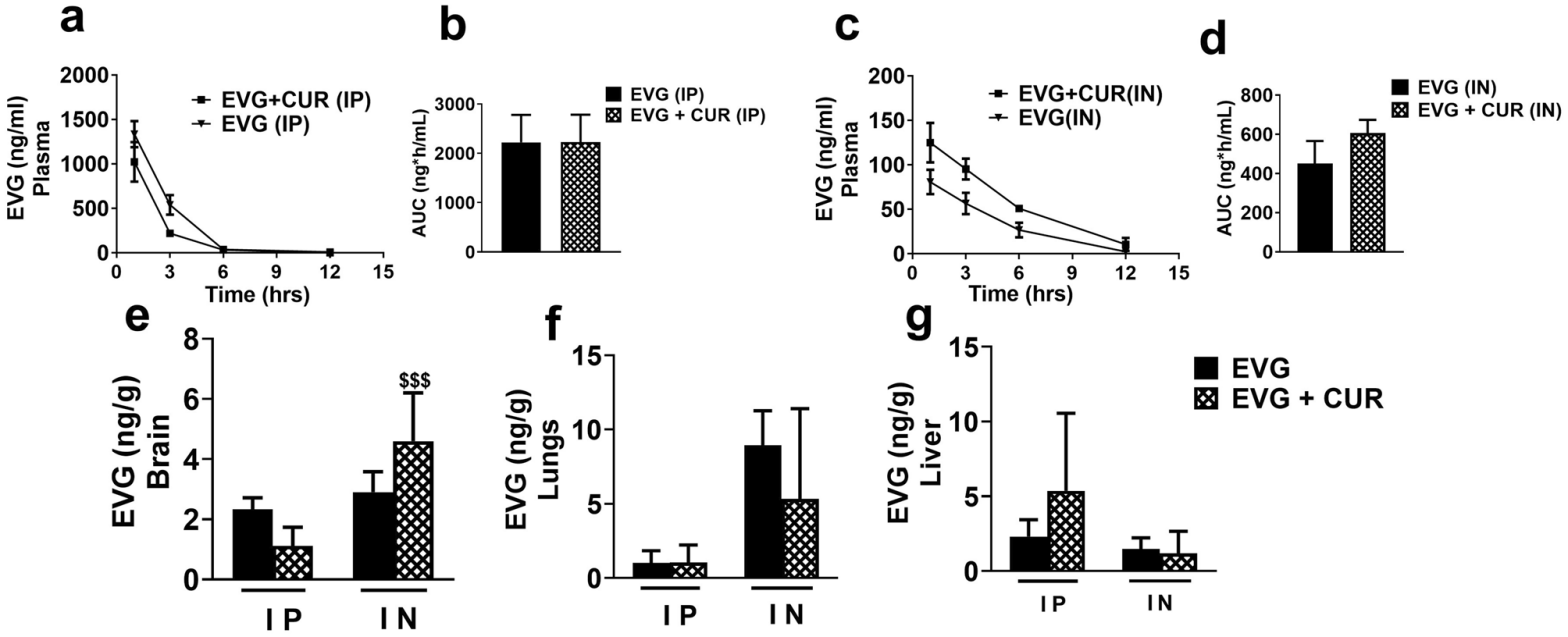
Biodistribution of EVG (5 mg/kg) in presence of CUR (4 mg/kg) administered via IP and IN in Balb/c mice. The concentrations of EVG were measured in plasma (**a**, **c**) at 1, 3, 6, and 12 h and in the brain (**e**), lungs (**f**), and liver (**g**) at 12 h. Concentrations are expressed as ng/g in tissues and ng/ml in plasma. Statistical analysis was performed using ANOVA (multiple comparisons) and t-test (two groups). Results are expressed as means ± S.E.M (n = 6). $$$ represents *p* ≤ 0.001 when compared to EVG + CUR (IP).

This study was performed to evaluate the in vivo biodistribution of EVG (5 mg/kg) in the presence of CUR (4 mg/kg) at low dose using both the IP and IN routes (Fig. 2a–g). The results indicate that the IP or IN administration of EVG in the presence of CUR does not lead to an increased concentration of EVG in plasma (Fig. 2a–d). However, like high dose study, the results indicated that CUR addition to combination treatment significantly increases EVG accumulation in the brain upon IN administration when compared to EVG + CUR (IP) group (Fig. 2e, $$$*p* ≤ 0.001). The biodistribution of EVG in the lungs and liver did not exhibit any significant alterations upon the addition of CUR in the treatment, regardless of the administration route (IP or IN) (Fig. 2f and g). Overall, although low dose treatment was less effective in increasing EVG concentrations in combination therapy in plasma or organs, it was still effective via IN compared with IP route in brain.

### Effect of CUR on neural marker proteins in EVG treated mice

**Figure 3:**
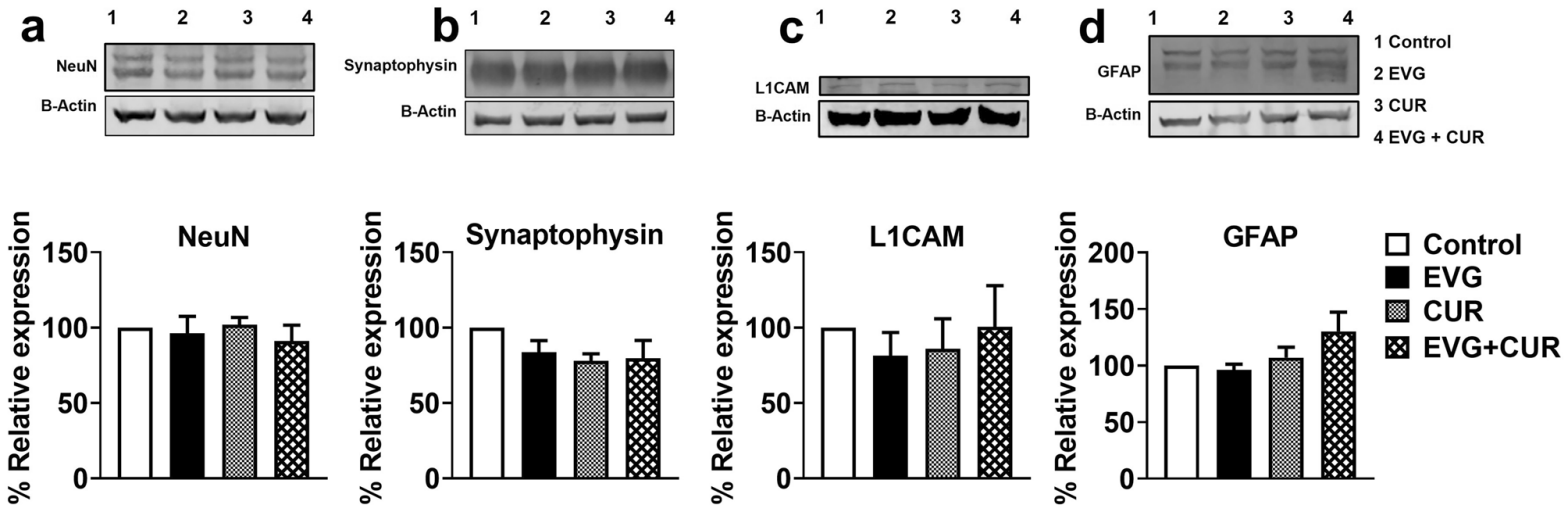
Western blot analysis of neural protein markers upon treatment with EVG (25 mg/kg), and CUR (20 mg/kg) in Balb/c mice brain. Protein was isolated from treated mice and expression of (**a**) NeuN, (**b**) Synaptophysin, (**c**) L1CAM, (**d**) GFAP was analyzed using western blotting. Results are expressed as means ± S.E.M (*n* = 4). Statistical analysis was performed using one-way ANOVA with Tukey’s post-hoc test for multiple group comparisons.

HAND involves key events such as neuronal apoptosis, dysregulation of neuronal support cells, and loss of dendritic arbor. Therefore, we aimed to examine whether specific neuronal markers (synaptophysin, L1CAM, NeuN, and GFAP) in mice brains are altered when treated with high dose IN EVG + CUR (25 and 20 mg/kg, respectively) for 12 h. Our findings showed that neither EVG or CUR alone nor addition of CUR to the EVG treatment altered the expression of these neuronal markers compared to the control group (Fig. 3a–d; Supplementary Fig. S1). This suggests that the neural homeostasis is not affected by the addition of CUR to the EVG treatment, at least upon acute exposure.

### Effect of CUR on intracellular EVG concentration in U1 macrophages

**Figure 4:**
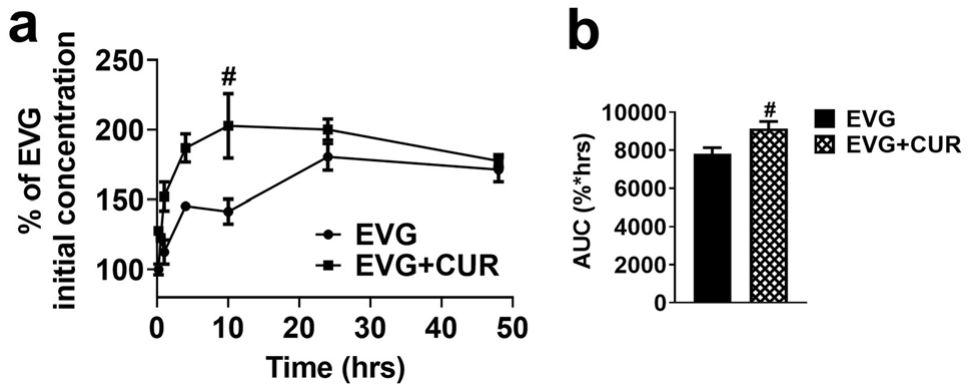
Accumulation of EVG in U1 macrophages over time upon treatment with EVG (1 µM) and CUR (5 µM). (**a**) Concentration–time profiles depicting the levels of EVG. (**b**) Calculation of the area under the curve (AUC) for EVG and EVG + CUR. Results expressed as means ± SEM (*n* = 4). Statistical analysis was performed using a t-test. # represents *p* ≤ 0.05 when compared to EVG.

In our 48 h experiment involving HIV-infected U1 macrophages, we administered EVG (1 µM) either alone or in combination with CUR (5 µM), and the intracellular EVG levels were measured (represented as percentage of the initial concentration). Our results consistently demonstrated higher intracellular EVG concentrations in the EVG + CUR group compared to the EVG only group (Fig. 4a, #*p* ≤ 0.05 at 10 min). Moreover, to assess the overall exposure to EVG, we calculated the total area under the curve (AUC_tot_) for EVG in both treatment groups. Notably, the AUC_tot_ of EVG + CUR was significantly higher than that of the EVG only group (Fig. 4b, #*p* ≤ 0.05). These findings highlight the ability of CUR to enhance the intracellular delivery and retention of EVG in U1 macrophages, which could subsequently enhance EVG efficacy.

### Effects of CUR on oxidative stress and antioxidant capacity in EVG-treated U1 macrophages

**Figure 5:**
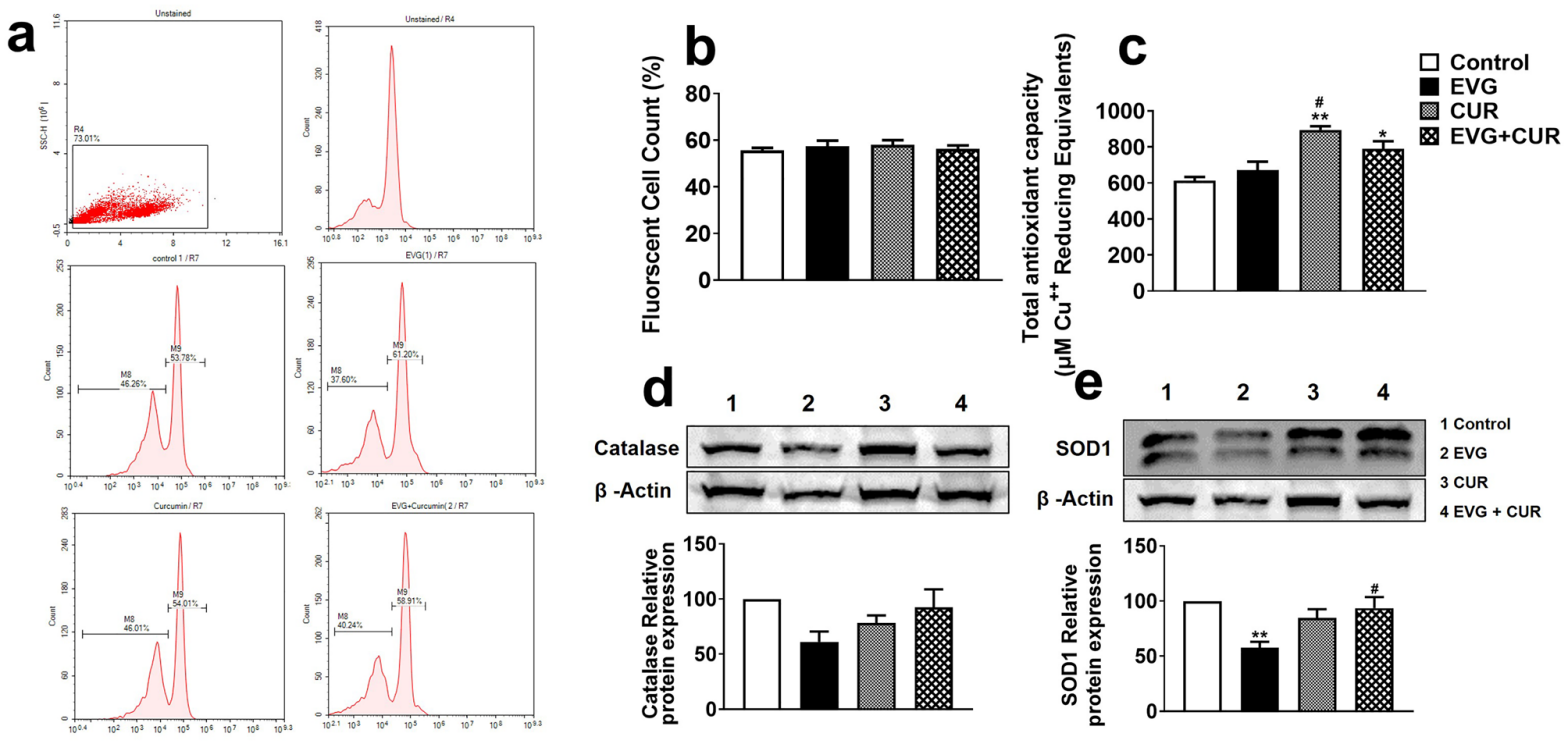
Effects of EVG and CUR on reactive oxygen species (ROS), antioxidant capacity and anti-oxidant enzymes in U1 macrophages. U1 macrophages were treated with EVG (1 µM) and CUR (5 µM) for one day, and (**a**, **b**) ROS levels were assessed using CM-DCFDA dye and flow cytometry (excitation/emission: 495/519 nm). (**c**) Total antioxidant capacity of the cells was measured using the Total Antioxidant Capacity Assay Kit, and the Y-axis represents the total reduced Cu + in µM as a measure of antioxidant capacity. **(d**, **e**) Protein levels of antioxidant enzymes (catalase and SOD1) were evaluated in U1 macrophages using Western blot analysis. Statistical analysis was performed using one-way ANOVA with Tukey’s post-hoc test. Results are expressed as means ± S.E.M (*n* = 4). * and ** represent *p* ≤ 0.05 and *p* ≤ 0.01, respectively, when compared to control. # represents *p* ≤ 0.05 when compared to EVG.

To determine whether the addition of CUR to the EVG treatment results in alteration of oxidative stress, we measured the reactive oxygen species (ROS) levels in the EVG + CUR treated U1 macrophages. Our results showed that 24 h treatment with EVG + CUR does not alter the ROS levels when compared to control treatment (Fig. 5a, b). While the biological effects resulting from ROS-induced oxidative stress are widely utilized for monitoring purposes, it is equally crucial to assess the antioxidant capacity of biological fluids and cells. In this study, we assessed the overall antioxidant capacity of U1 macrophages. The findings revealed that CUR alone significantly elevated the antioxidant capacity in comparison to the control group (Fig. 5c, **p ≤ 0.01). Moreover, the combination of CUR with EVG also enhanced the antioxidant capacity compared to the control (Fig. 5c, *p ≤ 0.05). Furthermore, we examined the protein expression of two important antioxidant enzymes, catalase and SOD1, in U1 macrophages after 24 h exposure to EVG + CUR. Our findings revealed that EVG treatment alone led to a significant decrease in SOD1 expression, and a similar trend was observed with catalase expression (Fig. 5d and e, **p ≤ 0.01; Supplementary Fig. S2). However, the addition of CUR to the EVG treatment resulted in increased SOD1 expression (Fig. 5e, #*p* ≤ 0.01). Although the trend for catalase expression followed a similar pattern with EVG and CUR treatment, it did not reach statistical significance. Taken together, the results suggest that although EVG alone increases/shows no difference in oxidative stress or antioxidant capacity, CUR alone and in the presence of EVG increases antioxidant capacity and perhaps decreases oxidative stress.

### Effect of CUR on modulation of EVG-induced cytokine profile in U1 macrophages

**Figure 6:**
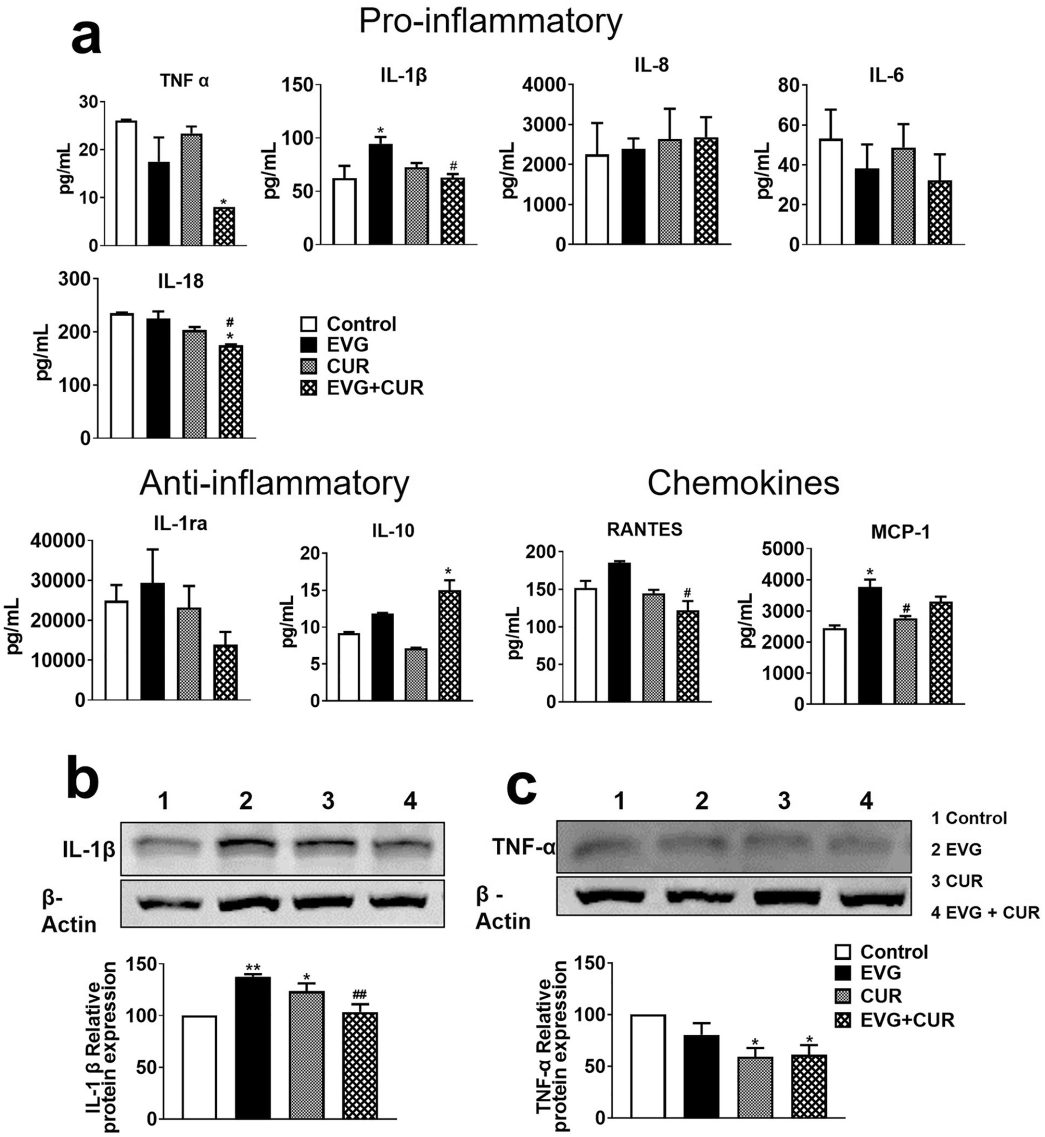
Modulation of cytokines and chemokines in U1 macrophages by EVG in the presence of CUR. U1 macrophages were treated with control (DMSO), EVG (1 µM), and CUR (5 µM) for one day, and (**a**) the protein levels of various cytokines and chemokines were measured in the culture media using Human Custom Procartaplex 9-plex assay. (**b**) The expression of IL-1β and TNF-α proteins in U1 macrophages was assessed by Western blot analysis. Statistical analysis was conducted using one-way ANOVA with Tukey’s post-hoc test. Results are expressed as means ± S.E.M (*n* = 4). * and ** represent *p* ≤ 0.05 and *p* ≤ 0.01, respectively, when compared to control and # and ## represent *p* ≤ 0.05, and *p* ≤ 0.01, respectively, when compared to EVG alone.

In this study, the effect of combining CUR with EVG on the release of proinflammatory cytokines (IL-1β, TNFα, IL-6, IL-8, IL-18), anti-inflammatory cytokines (IL-10, IL-1RA), and chemokines (RANTES, MCP-1) was evaluated in the media released from U1 macrophages. The results indicated that the addition of CUR to EVG treatment significantly reduced the level of the proinflammatory cytokine TNFα (Fig. 6a, *p ≤ 0.05) when compared to the control group. Furthermore, the results showed that the level of IL-1β was elevated in EVG-only (Fig. 6a, *p ≤ 0.05) treatment but significantly decreased in the EVG + CUR (Fig. 6a, #*p* ≤ 0.05) treatment group. Additionally, CUR significantly lowered the level of IL-18 (Fig. 6a, *p ≤ 0.05, #*p* ≤ 0.05) when compared to both the control group and the EVG-only treatment group. However, we noticed no statistical difference in the levels of IL-6 and IL-8 in all treatment groups when compared to control. The level of anti-inflammatory cytokine IL-10 (Fig. 6a, *p ≤ 0.05) was increased in EVG + CUR treatment group compared to control. However, IL-1ra showed no significant changes in any treatment groups compared to control. With respect to the chemokines, RANTES and MCP-1, both showed increased levels by EVG-only treatment (Fig. 6a, *p ≤ 0.05 for MCP-1). However, CUR alone (Fig. 6a, #*p* ≤ 0.05 for MCP-1) or combination (Fig. 6a, #*p* ≤ 0.05 for RANTES) showed decreased levels of chemokines. Taken together, relatively decreased levels of proinflammatory cytokines and chemokines and increased levels of anti-inflammatory cytokines by EVG + CUR compared to control and/or EVG-only suggest that the combination therapy can decrease systemic inflammatory response in brain macrophages.

Furthermore, to validate the results obtained on cytokines released in the media (systemic inflammation), we also measured the effect of CUR + EVG treatment on cellular cytokines (IL1-β and TNF-α) using Western blot. TNF-α and IL-1β play a major role in HIV pathogenesis. Our findings demonstrated that treatment with EVG alone significantly increased IL-1β levels compared to the control group (Fig. 6b, **p ≤ 0.01; Supplementary Fig. S3). However, the addition of CUR to the EVG treatment effectively reversed this increase in IL-1β expression (Fig. 6b, ##*p* ≤ 0.01). Regarding TNF-α, both CUR treatment alone and EVG + CUR (Fig. 6c, *p ≤ 0.05; Supplementary Fig. S3) (Fig. 6c, *p ≤ 0.05) resulted in decreased levels compared to the control treatment. These results further suggest that CUR alone or combination reduces cellular inflammatory response.

## Discussion

The findings of our study address the challenges associated with drug delivery across the BBB in context to HIV neuropathogenesis. The limited penetration of ART drugs into the CNS leads to persistent viral replication and neuroinflammation, contributing to neuronal damage^4^. To overcome this barrier, prior research has explored innovative strategies for enhancing CNS drug delivery. One approach is the development of nanocarriers, such as the poloxamer-PLGA nanocarrier used by Yuqing et al., which demonstrated successful transmigration across an in vitro BBB model and improved viral suppression in HIV-infected macrophages^25^. This highlights the potential of nanocarriers in improving drug delivery to the CNS. Furthermore, studies have shown the role of efflux transporters, particularly P-gp, in limiting the intracellular accumulation of antiretroviral drugs^7^. Variations in P-gp expression among macrophage subsets and HIV-infected patients have been observed, potentially impacting drug concentrations and HIV replication^26^. Inhibition of P-gp function, as demonstrated by Jiu-Cong Zhang et al. using CUR, may enhance the intracellular concentration and overall exposure of antiretroviral drugs^27, 28^.

In our previous study, we investigated the IN delivery of darunavir (DRV) to improve brain drug concentration in mice^29^. We compared the biodistribution of DRV at high (25 mg/kg) and low (2.5 mg/kg) concentrations using IV and IN routes in the brain, liver, lungs, and plasma. Our results showed that IN administration significantly increased DRV penetration in the brain compared to IV administration, at both low (5 mg/kg) and high (25 mg/kg) concentrations. Furthermore, IN administration resulted in lower DRV concentrations in plasma and liver compared to IV administration. These findings indicate that the IN route can enhance the concentration of DRV in the brain, potentially suppressing HIV in brain reservoirs, while reducing off-target effects in peripheral organs^29^.

In the present study, we investigated the potential of CUR as an adjuvant to improve the delivery of the ART drug EVG across the BBB and reduce pathogenesis associated with HIV such as oxidative stress and inflammatory response in macrophages, an important CNS viral reservoir. Our results demonstrated that the addition of CUR increased the intracellular concentration of EVG in U1 macrophages, suggesting improved efficacy. Moreover, in vivo experiments using mouse models showed that CUR enhances the brain delivery of EVG, particularly when administered through IN route. The IN route provides a non-invasive mode of drug delivery to the brain by bypassing the BBB^29^. This route takes advantage of the direct nose-to-brain pathway, resulting in faster onset of action and improved drug delivery to specific brain regions. The finding is consistent with our previous findings with DRV delivery via IN route. Thus, our findings support the potential of IN administration of CUR-adjuvanted EVG as an effective approach for enhancing CNS drug delivery. Furthermore, CUR may enhance EVG delivery by inhibiting CYP3A4 and/or P-gp activities. Our speculation is based on the literatures that suggest that inhibition of CYP3A4 and P-gp by CUR enhances the accumulation etoposide and tamoxifen in various systems^30, 31^. Thus, it’s likely that CUR increases EVG cellular concentration in U1 macrophages by inhibiting CYP3A4 and/or P-gp. We also suggest that CUR might improve EVG delivery across the blood–brain barrier and into macrophages via inhibition of P-gp, thereby enhancing its efficacy However, other mechanisms for CUR-mediated increase in EVG concentrations cannot be ruled out.

The inefficiency of current ART regimens in treating HAND can be attributed to their limited ability to target the inflammatory cascades associated with neuroHIV^32^. HIV-infected individuals receiving ART have been reported to exhibit higher levels of free radical species compared to untreated HIV-positive individuals or healthy subjects^33^. This suggests that HIV infection itself, along with the introduction of ART, may induce oxidative stress and exacerbate HIV pathogenesis. Various intracellular antioxidant defenses play a crucial role in detoxifying ROS and protecting cells. Enzymes such as SOD1 and catalase contribute to the quenching and conversion of ROS into harmless byproducts^34^. However, the balance between ROS production and antioxidant defenses can be disrupted in HIV-infected individuals^33^. CUR, with its well-documented anti-inflammatory and antioxidant properties, is a promising candidate for adjuvant therapy in HIV treatment^35^. It inhibits key enzymes involved in HIV replication, such as protease and integrase, and targets the NF-κB pathway, which is essential for HIV gene expression^35, 36^. CUR acts as a bifunctional antioxidant by directly scavenging ROS and inducing an antioxidant response^37^. These properties make CUR an attractive option for enhancing the efficacy of HIV treatment.

Oxidative stress and inflammation are closely linked processes. The accumulation of ROS triggers oxidative stress, which in turn enhances inflammation by activating transcription factors associated with inflammation. CUR can reduce ROS production by affecting NADPH oxidase and increasing the activity of antioxidant enzymes^34^. Additionally, CUR’s modulation of the Nrf2-Keap1 pathway contributes to its anti-inflammatory and antioxidant effects^38^. In our study, we observed that the combination of CUR and EVG had beneficial effects on modulating oxidative stress and antioxidant capacity in U1 macrophages. While EVG alone showed no significant changes, CUR alone and in combination with EVG reduced oxidative stress and increased antioxidant capacity. These findings underscore the potential neuroprotective effects of CUR and its ability to modulate oxidative capacity in the context of HIV infection.

Initiating ART even during the early viremic phase of HIV infection may not completely reverse all HIV-induced immune alterations^39^. Despite the accessibility and effectiveness of ART, achieving full immune restoration and reducing immune activation in HIV-infected individuals remains challenging^40^. Several factors contribute to persistent immune activation even with virologic suppression, including ongoing low-level HIV replication in tissue reservoirs, the presence of viral proteins triggering immune responses, inflammatory lipids, co-infections, and damage to the gastrointestinal tract leading to microbial translocation^41^. These mechanisms collectively contribute to sustained immune dysfunction and inflammation in ART-treated HIV infection. Hemalatha et al.'s study highlighted significant differences in inflammatory biomarkers between people living with HIV (PLHIV) on long-term ART and healthy individuals^42^. These biomarkers are associated with inflammatory conditions observed in various diseases such as cancer, cardiovascular diseases, neurological disorders, and skeletal diseases. These findings suggest that PLHIV, even with successful ART, may have an increased risk of developing inflammatory diseases and experiencing inflamm-aging^42^. Therefore, interventions such as adjuvant therapy with anti-inflammatory agents like CUR could be considered to reduce excessive immune activation and inflammation in PLHIV. Preclinical studies on inflammatory cells and animal models have demonstrated the ability of CUR to decrease levels of pro-inflammatory mediators such as interleukins (IL-1, IL-1β, IL-6, IL-8, IL-17, IL-27), tumor necrosis factor-α (TNF-α), inducible nitric oxide synthase (iNOS), regulated upon activation normal T cell expressed and secreted factor (RANTES), granulocyte colony-stimulating factor (G-CSF), and monocyte chemotactic protein-1 (MCP-1)^18^. Clinical trials have also shown that CUR can reduce inflammatory mediators such as C-reactive protein (CRP) and TNF in PLHIV^43^.

In our study, the combination of CUR and EVG has demonstrated promising results. The addition of CUR to EVG treatment led to a significant decrease in proinflammatory cytokines (IL-1β, TNFα, IL-18) and chemokines (RANTES, MCP-1) in cell-based experiments. In HAND, key events involve neuronal apoptosis, dysregulation of neuronal support cells, and dendritic arbor loss. To explore neural markers, we examined synaptophysin, L1CAM, NeuN, and GFAP in mouse brains. NeuN, a splicing regulator, shows increased cytoplasmic localization in HIV-associated neurocognitive disorders. GFAP, an astrocyte marker, is upregulated in reactive astrocytes. Synaptophysin, a synaptic vesicle regulator, is abundant in synaptic transmission. L1CAM plays a role in neural development and regeneration. Our study found that CUR addition to EVG treatment did not significantly alter the expression of these neuronal markers, indicating preserved neural homeostasis.

In conclusion, the findings of our study, along with the additional evidence presented, highlight the potential benefits of incorporating CUR as an adjuvant therapy in the treatment of HIV neuropathogenesis. The combination of CUR with the antiretroviral drug EVG has shown improvements in total antioxidant activity and a reduction in inflammation, as demonstrated by the modulation of cytokine profiles in an in vitro HIV setting. Further investigation using HIV-infected primary macrophages and HIV mouse models would provide valuable insights into the implications of CUR on HIV neuropathogenesis. Additionally, development of EVG nanoparticles using poloxamer-PLGA by our research group has demonstrated significant increase in brain EVG concentrations and subsequent HIV suppression. Therefore, formulating CUR along with EVG in PLGA nanoparticles can further enhance its adjuvant profile and improve the overall outcomes in the treatment of HIV neuropathogenesis.

## Materials and methods

### Materials

Elvitegravir (EVG, E509000) was obtained from Toronto Research Chemicals, Inc. (Ontario, Canada). Sterile phosphate-buffered saline (PBS) (10100- 031) was sourced from Gibco (Dublin, Ireland). LC/MS-grade acetonitrile (A955) and formic acid (AC270480010), BD PrecisionGlide 25G needle (14-826-49), and BD 1 Ml TB syringe (14-826-88) were procured from Fisher Scientific (Hampton, NH, USA).

### Animals

Male and female Balb/c mice, aged 10 to 12 weeks, were obtained from Jackson Laboratory (Bar Harbor, MA) and allowed to acclimate in the animal facility for a minimum of 7 days before the start of the study. The mice were housed in groups of five per cage in a sterile room with a 12/12-h light–dark cycle. The room maintained a constant temperature and humidity, and the mice had ad libitum access to food and water throughout the study. All animal procedures were approved by the institutional animal care and use committee of the University of Tennessee Health Science Center (UTHSC-IACUC protocol #20-0165) and were performed in accordance with the Guide for the Care and Use of Laboratory Animals from the National Institutes of Health. All methods with animal studies were reported in accordance with ARRIVE guidelines. For the EVG biodistribution study, a total of 96 mice were randomly divided into two groups for IP and IN administration. Each group was further divided into four subgroups corresponding to four time points (1, 3, 6, and 12 h), with three females and three males in each subgroup for both EVG and EVG + CUR treatment. The chosen time points for our analysis were based on EVG’s pharmacokinetics and biodistribution. We measured plasma concentrations at 1, 3, 6, and 12 h due to the drug's exponential decline within this timeframe, which was practically undetectable after 12 h. In the brain and other organs, we set the terminal time point at 12 h. In the low-dose study, mice were administered EVG (5 mg/kg) and CUR (4 mg/kg), while in the high-dose study, EVG (25 mg/kg) and CUR (20 mg/kg) were administered. These concentrations were determined based on previous studies and literature sources^29, 44^. The drugs were dissolved in a solution containing 5% DMSO, 80% PEG400, and 15% PBS. For IN administration, the concentration of EVG in the final solution was adjusted to 0.5 ml/kg in mice. The final volume of DMSO used was 0.025 ml/kg, which was below the reported non-toxic dose. Mice were euthanized under deep isoflurane anesthesia followed by cervical dislocation, and blood samples were collected at the designated time points (1, 3, 6, and 12 h) via cardiac puncture using EDTA-containing blood-collection tubes. The blood samples were allowed to settle at room temperature and then centrifuged at 6000 rpm for 10 min at 4 °C to obtain plasma. Tissues, including the brain, liver, and lungs, were collected at the terminal time point of 12 h. Plasma and brain samples were stored in tubes and frozen at -80 °C until further analysis using LC–MS/MS. Tissue samples were homogenized in 1X phosphate-buffered saline (PBS) at a ratio of 1:4 (wt/vol). Fifty µL of each plasma and tissue sample was used for LC–MS/MS analysis, which was performed following established protocols using appropriate LC–MS/MS equipment and methodologies.

### Cell culture and treatment

U1 cells, a chronically HIV-infected U937 cell line, were obtained from the NIH AIDS Reagent Program (Germantown, MD). U1 cells are the major HIV model cells to study in vitro HIV-associated pathogenesis including oxidative stress and inflammatory response^45, 46^. The data obtained with U1 macrophages are correlated with human primary monocyte-derived macrophages^47^. The U1 cells were cultured in RPMI 1640 media supplemented with 10% fetal bovine serum (FBS) and 1% L-glutamine. To differentiate the U1 cells into macrophages, 0.3 million cells in 0.4 ml of media containing 100 nM phorbol 12-myristate 13-acetate (PMA) were seeded in each well of a 12-well plate. After 3 days of differentiation, the media was aspirated, and the cells were washed with PBS before adding fresh media to the differentiated cells. The cells were then incubated for 3–4 h before starting the treatment. The differentiated U1 macrophages were subjected to different treatment conditions. This included a control group treated with DMSO, as well as experimental groups treated with EVG (1 µM), CUR (5 µM). These EVG and CUR concentrations, which are near physiological, were chosen based on our previous study and studies from other group^29, 44, 48^. The cells were exposed to the respective treatments for a defined period as per the treatment protocol of each assay. After the treatment duration, the U1 macrophages were harvested for further analysis. The cells were collected and processed for downstream experiments as per the specific requirements of each assay.

### Quantification of intracellular ROS with fluorescence-based assay

To quantify the ROS level, we used flow cytometry analysis along with the fluorescence dye CM-H2DCFDA (ThermoFisher Scientific) as described before^49^. After thoroughly washing the treated cells with PBS, they were resuspended in 5 μM of CM-H2DCFDA in PBS and incubated in the dark at room temperature for 45 min. Following the incubation, the cells were washed and resuspended in 300 μL of PBS. The ROS produced in the cells was then detected and analyzed using the built-in flow cytometer software (Agilent NovoCyte).

### Total antioxidant capacity

The antioxidant capacity of U1 cells treated with EVG + CUR was determined using the Total Antioxidant Capacity Assay (TCA) Kit (Cell Biolabs, San Diego, CA, USA) according to the manufacturer's instructions. The assay quantifies the antioxidant capacity by measuring the copper reducing equivalents (CRE) in the samples. The results are reported as μM CRE, which is indicative of the total antioxidant capacity of the samples.

### Cytokine analysis

The levels of various cytokines and chemokines, including pro-inflammatory cytokines IL-1β, TNF-α, IL-8, IL-6, IL-18; anti-inflammatory cytokines IL-1RA, IL-10; and chemokines MCP-1 and RANTES, were measured from the culture media of differentiated U1 macrophages and mice plasma. The measurements were performed using Human Custom Procartaplex 9-plex and Mouse Custom Procartaplex 6-plex (Invitrogen, ThermoFisher Scientific, Grand Island, NY, USA), following the manufacturer’s protocol as previously described^45^. Samples, standards, and magnetic beads were added to a 96-well ELISA plate and mixed thoroughly on a plate shaker for 1 h at room temperature, followed by overnight incubation at 4 °C. The beads were then washed, and the detection antibody, streptavidin-PE, and reading buffer were added, with subsequent washing steps after each addition. The concentration of cytokines and chemokines (pg/mL) was measured using a Magpix system, and the data were analyzed using the xPONENT® software.

### Western blotting

Protein expression in the treated cells and mouse brain was determined using Western blotting. For the evaluation of IL-1β, TNF-α, catalase, and SOD1 expression in EVG + CUR treated U1 cells, an equal amount of protein (15 μg) was used from control (DMSO), EVG, CUR, and EVG + CUR treated differentiated U1 macrophages. Similarly, for the evaluation of neural marker proteins NeuN, Synaptophysin, L1CAM, and GFAP in EVG + CUR treated mice, an equal amount of protein (15 μg) was used from control (DMSO), EVG, CUR, and EVG + CUR treated mouse brain homogenate. The proteins from different study groups were loaded onto a polyacrylamide gel, with a stacking gel concentration of 4% and a resolving gel concentration of 10%. The gel was run for 90 min at 150 V, and then the proteins were transferred to a polyvinyl fluoride membrane using a current of 0.35 Amp for 90 min. After the transfer, the membrane was incubated with 5–10 mL of Li-Cor blocking buffer (LI-COR Biosciences, Lincoln, NE, USA) for 1 h to minimize nonspecific binding of antibodies. Subsequently, the membrane was incubated overnight at 4 °C with primary antibodies specific to the target proteins. The primary antibodies used were SOD1 mouse monoclonal antibody (1:1500 dilution, catalog no. sc-101523, Santa Cruz Biotechnology), CAT mouse monoclonal antibody (1:1000 dilution, catalog no. sc-365738, Santa Cruz Biotechnology), IL-1β rabbit polyclonal antibody (1:1000 dilution, catalog no. 16806-1AP, Proteintech), TNF-α rabbit polyclonal antibody (1:1000 dilution, catalog no. 16806-1AP, Proteintech), β-Actin rabbit polyclonal antibody (1:4000 dilution, catalog no. 20536-1-AP, Proteintech), NeuN rabbit polyclonal antibody (1:1000 dilution, catalog no. 26975-1-AP, Proteintech), Synaptophysin mouse monoclonal antibody (1:20,000 dilution, catalog no. 67864-1-Ig, Proteintech), β-Actin mouse monoclonal antibody (1:20,000 dilution, catalog no. 66009-1-Ig, Proteintech), GFAP rabbit polyclonal antibody (1:1000 dilution), and L1CAM rabbit polyclonal antibody (catalog no. 20659-1-AP, Proteintech Inc, Rosemont, IL, USA). The next day, the blots were washed three times with PBS containing 0.2% Tween-20 (PBST) and then incubated with the corresponding secondary antibodies, including Goat anti-Mouse Mab (1:10,000 dilution, LI-COR Biosciences) and Goat anti-Rabbit Mab (1:10,000 dilution, LI-COR Biosciences), for 1 h at room temperature in the dark. After another round of washing with PBST, the blots were scanned using Image Studio Lite version 4.0 in a Li-Cor Scanner (LI-COR Biosciences). Densitometric data were obtained from the Image Studio Lite software, and β-Actin was used as an internal loading control to normalize the expression of the proteins. All the original blots referenced in the document are provided in the supplementary file.

### Quantification of EVG using LC–MS/MS

The concentration of EVG in mouse plasma, tissue samples, and cell lysate samples was analyzed using our standardized LC–MS/MS method, as previously described^44^. The analytical system consisted of a Shimadzu liquid chromatographic system (Kyoto, Japan) coupled with an AB SCIEX Triple Quad 5500 tandem mass spectrometer (Framingham, MA). Separation of EVG was achieved using an Xterra® MS C18 column (125 Å, 3.5 μm, 4.6 mm × 50 mm; Waters, Milford, MA). The mobile phase consisted of two components: (A) water with 0.1% formic acid and (B) acetonitrile with 0.1% formic acid (v/v), flowing at a rate of 1 mL/min. A gradient elution was applied with 50% B for 0–1.5 min and 60% B for 1.5–5.1 min. The range of quantification for the assay was 1 to 500 ng/mL. EVG and the internal standard (IS) were eluted separately at approximately 3.27 and 2.72 min, respectively. Quantitative analysis was performed using multiple reactions monitoring (MRM) transitions: 447.9/343.8 for EVG and 721.3/296.1 for the IS. For EVG extraction from mouse plasma, livers, lungs, and brains, 4 volumes (200 μL) of cold acetonitrile for plasma and methanol for brains, both containing 50 ng/mL ritonavir (RTV) as an internal standard, were added to the samples. Media and cell lysate samples (50 μL) were mixed with 3 volumes of cold acetonitrile containing the internal standard (RTV, 50 ng/mL). The resulting solutions were vortexed and centrifuged at 10,000 rpm for 10 min to precipitate proteins. The clear supernatant was collected and subjected to analysis using the validated LC–MS/MS method. Calibration curves using control cell lysate, plasma, or tissue samples were prepared to account for matrix effects and ensure accurate quantification. To determine the intracellular concentration of EVG in U1 macrophages, a validated LC–MS/MS method with ritonavir (RTV) as an internal standard was employed. A calibration curve spanning a range of 50–2000 ng/mL was constructed using RIPA buffer to minimize matrix effects. The calibration curve exhibited excellent linearity (r^2^ = 0.999) with a weighting factor of 1/x^2^, enabling precise quantification of EVG concentration.

### Statistical analysis

The data obtained from the experiments were presented as mean ± standard error of the mean (SEM) and were derived from a minimum of three independent experiments. Statistical analyses were performed using GraphPad Prism version 9.5.1 for Windows (GraphPad Software, San Diego, California, USA, www.graphpad.com). The significance of differences between groups was determined using analysis of variance (ANOVA) with multiple comparisons or t-tests for comparisons between two groups, as appropriate. The level of significance was set at *p* ≤ 0.05 (Supplementary File 1).

### Supplementary Information

Supplementary Figures.

## Data Availability

All data generated or analyzed during this study are included in this published article (and its Supplementary Information files).
