# Supplementary material for: Curcumin enhances elvitegravir concentration and alleviates oxidative stress and inflammatory response
Source: Sci Rep. 2023 Nov 14;13:19864. doi: 10.1038/s41598-023-47226-1 (PMC10645974; doi:10.1038/s41598-023-47226-1)
Supplement: Supplementary file 1 — Supplementary Figures. [file 41598_2023_47226_MOESM1_ESM.pdf]

Title: Curcumin enhances elvitegravir concentration and alleviates oxidative stress and inflammatory response

Sandip Godse[1], Lina Zhou[1], Namita Sinha[1], Sunitha Kodidela[1], Asit Kumar[1], Udai P Singh[1] and Santosh Kumar[1*]

[1] Department of Pharmaceutical Sciences, College of Pharmacy, The University of Tennessee Health Science Center, Memphis, TN 38163, USA.

***Corresponding author:** Santosh Kumar, ksantosh@uthsc.edu

Supplementary Figure S1: Figure 3 original blots a, b, c, & d

Western blot analysis of neural protein markers upon treatment with EVG (25 mg/kg), and CUR (20 mg/kg) in Balb/c mice brain.

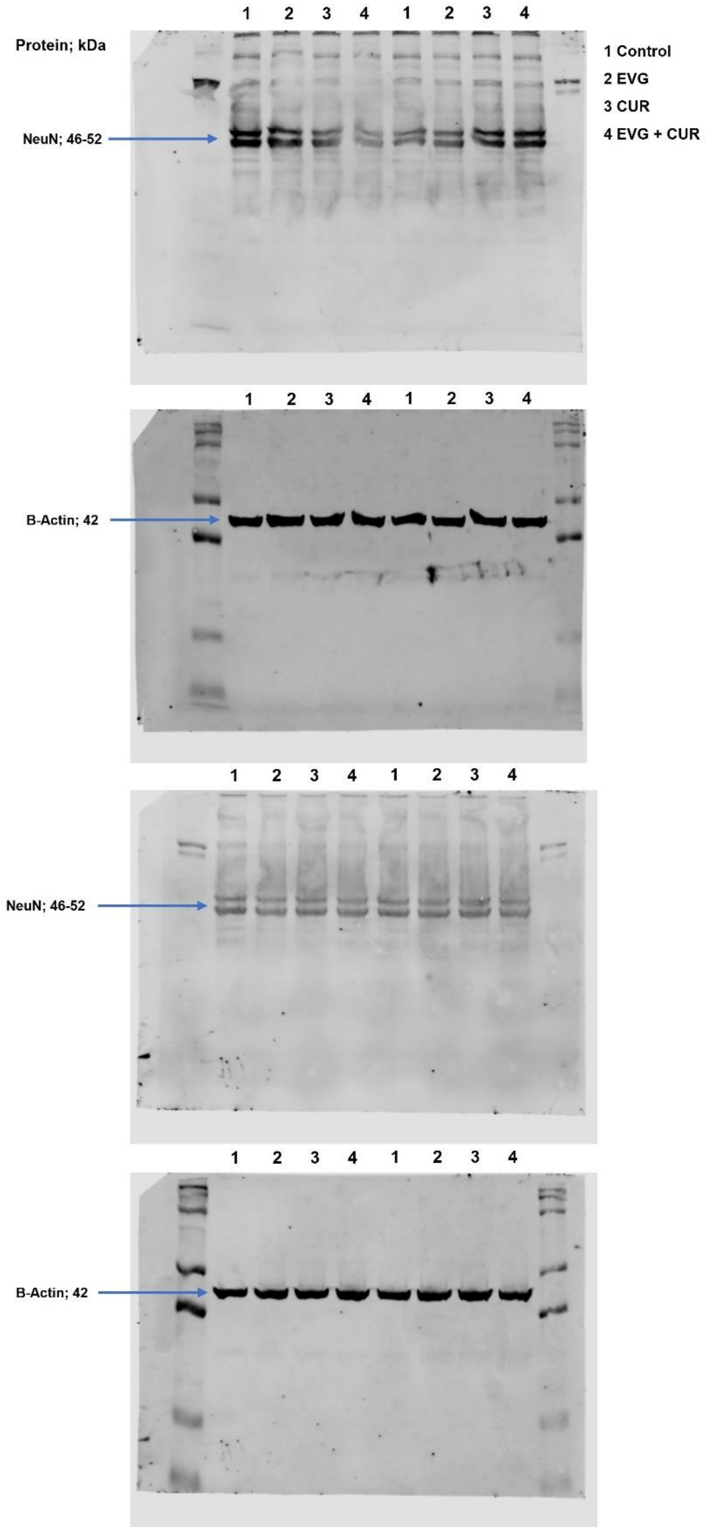

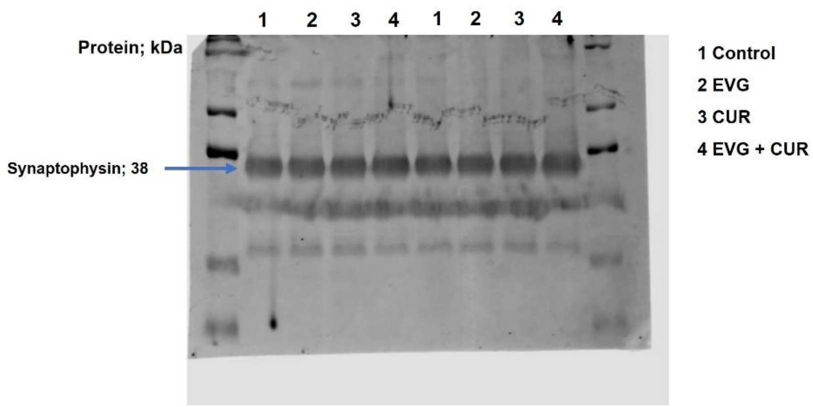
1 2 3 4 1 2 3 4
Protein; kDa
Synaptophysin; 38
1 Control
2 EVG
3 CUR
4 EVG + CUR

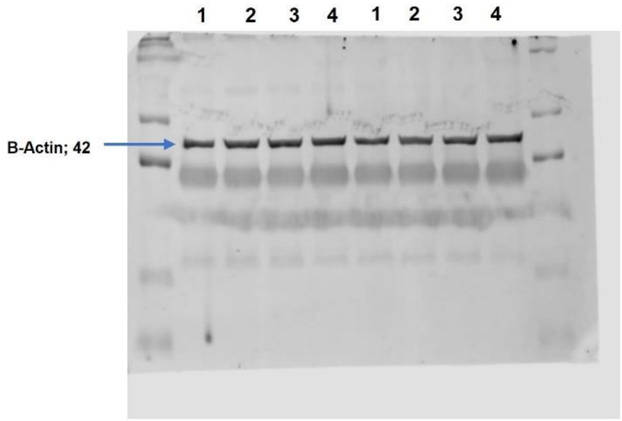
1 2 3 4 1 2 3 4
B-Actin; 42

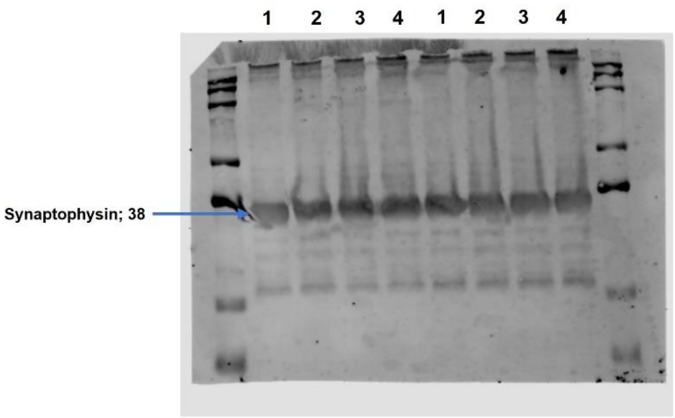
1 2 3 4 1 2 3 4
Synaptophysin; 38

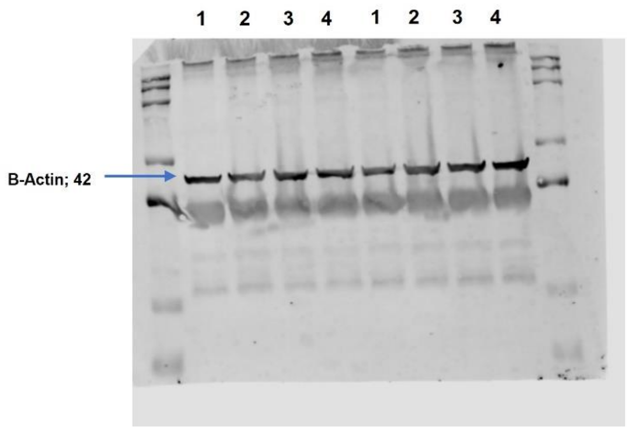
1 2 3 4 1 2 3 4
B-Actin; 42

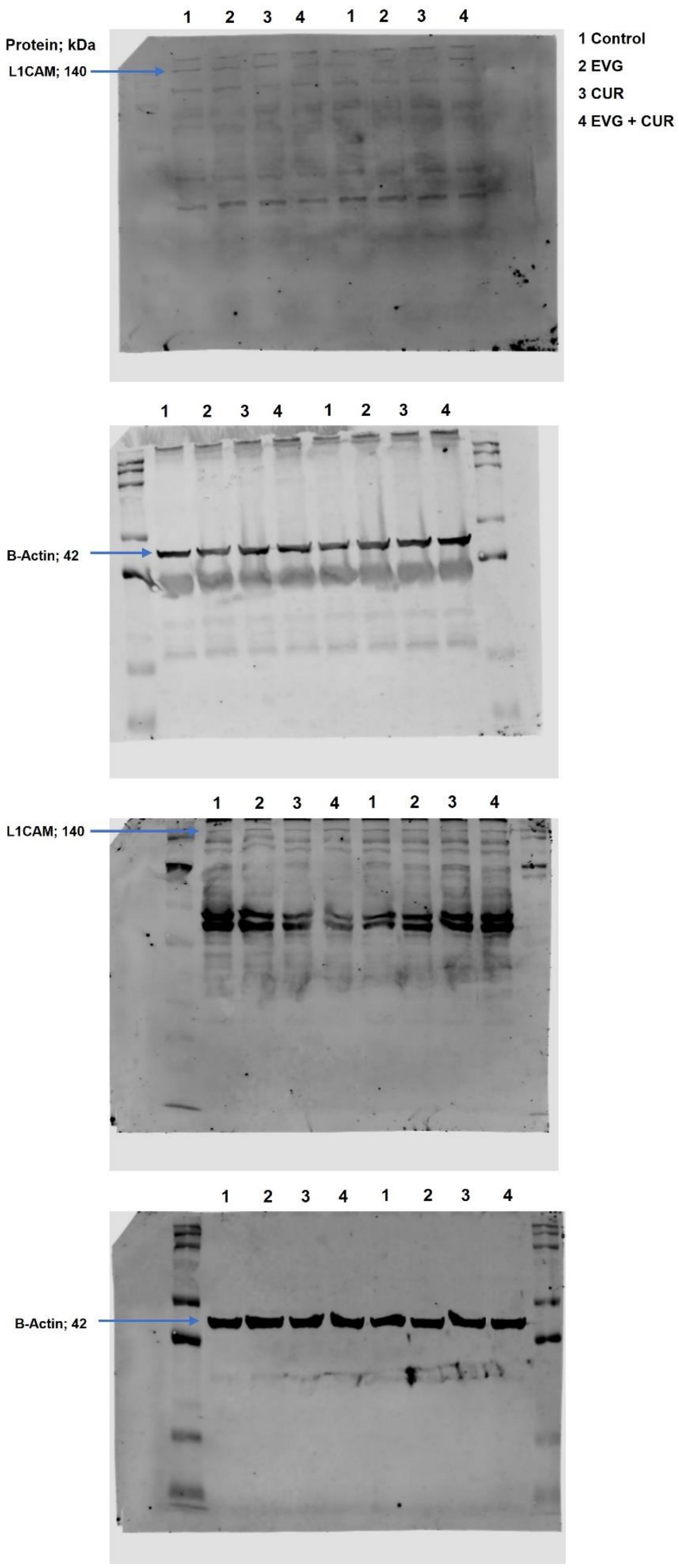
1 2 3 4 1 2 3 4
Protein; kDa
L1CAM; 140
1 Control
2 EVG
3 CUR
4 EVG + CUR
1 2 3 4 1 2 3 4
B-Actin; 42
1 2 3 4 1 2 3 4
L1CAM; 140
1 2 3 4 1 2 3 4
B-Actin; 42

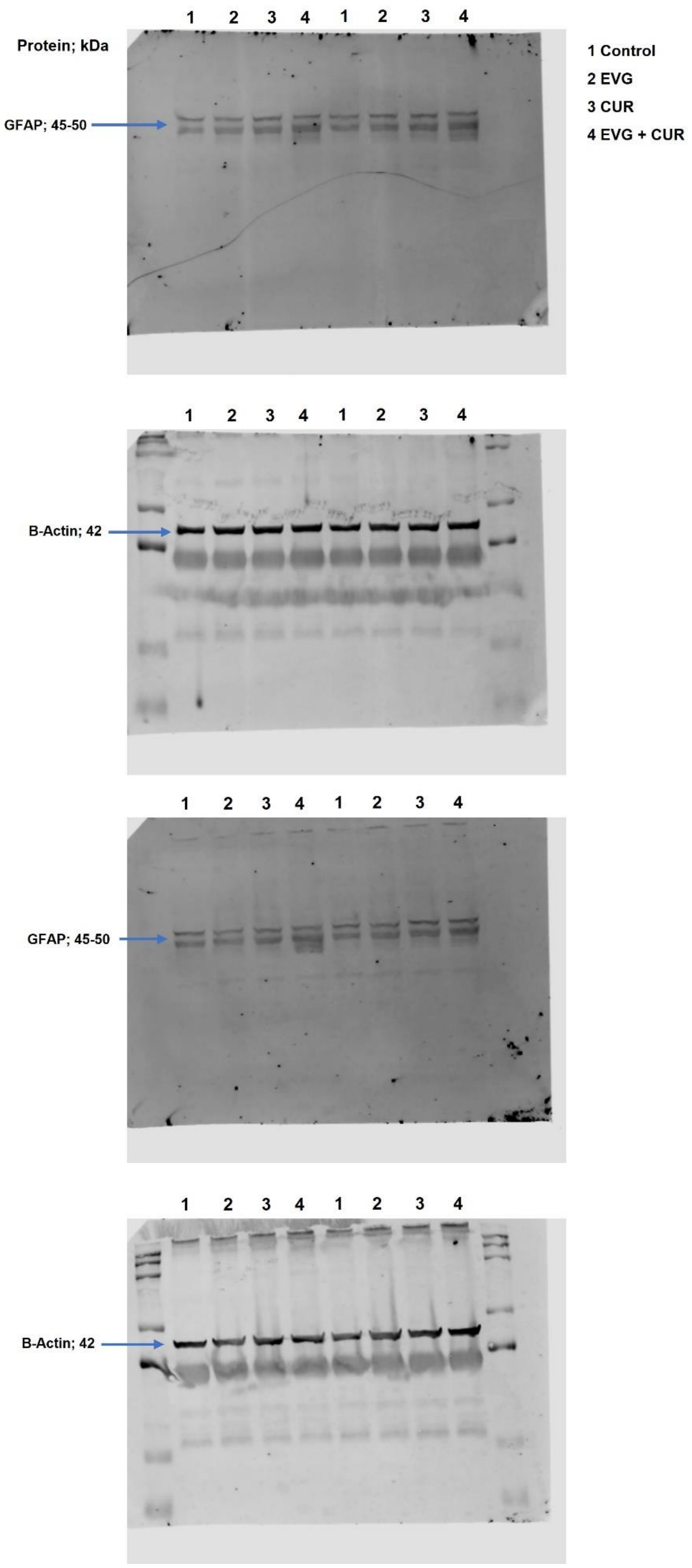
Protein; kDa
1 2 3 4 1 2 3 4
1 Control
2 EVG
3 CUR
4 EVG + CUR
GFAP; 45-50
1 2 3 4 1 2 3 4
B-Actin; 42
1 2 3 4 1 2 3 4
GFAP; 45-50
1 2 3 4 1 2 3 4
B-Actin; 42

Supplementary Figure S2: Figure 5 original blots d & e

Effect of EVG and CUR on antioxidant enzymes. Antioxidant enzymes (catalase and SOD1) proteins were measured in U1 macrophages by Western blot.

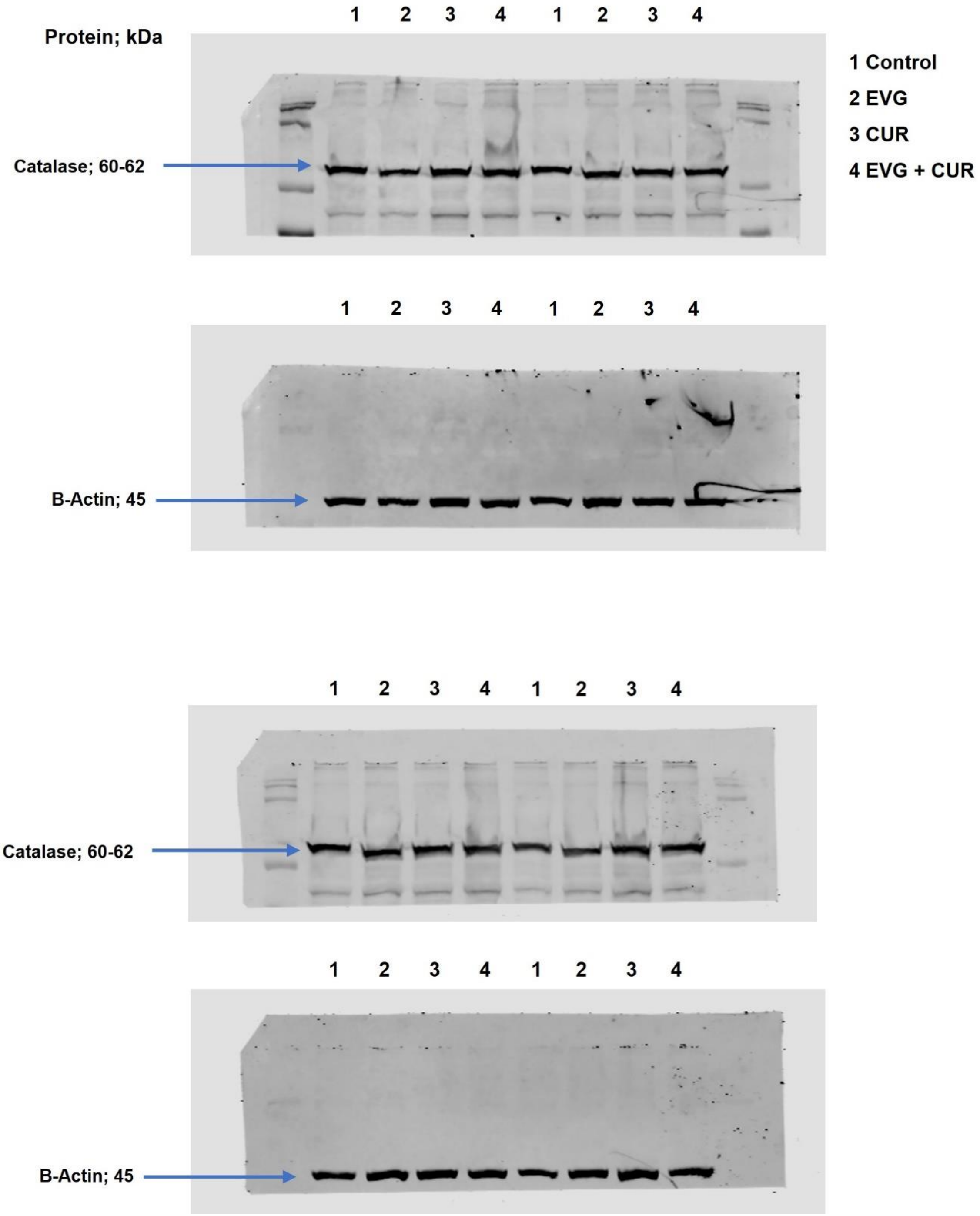

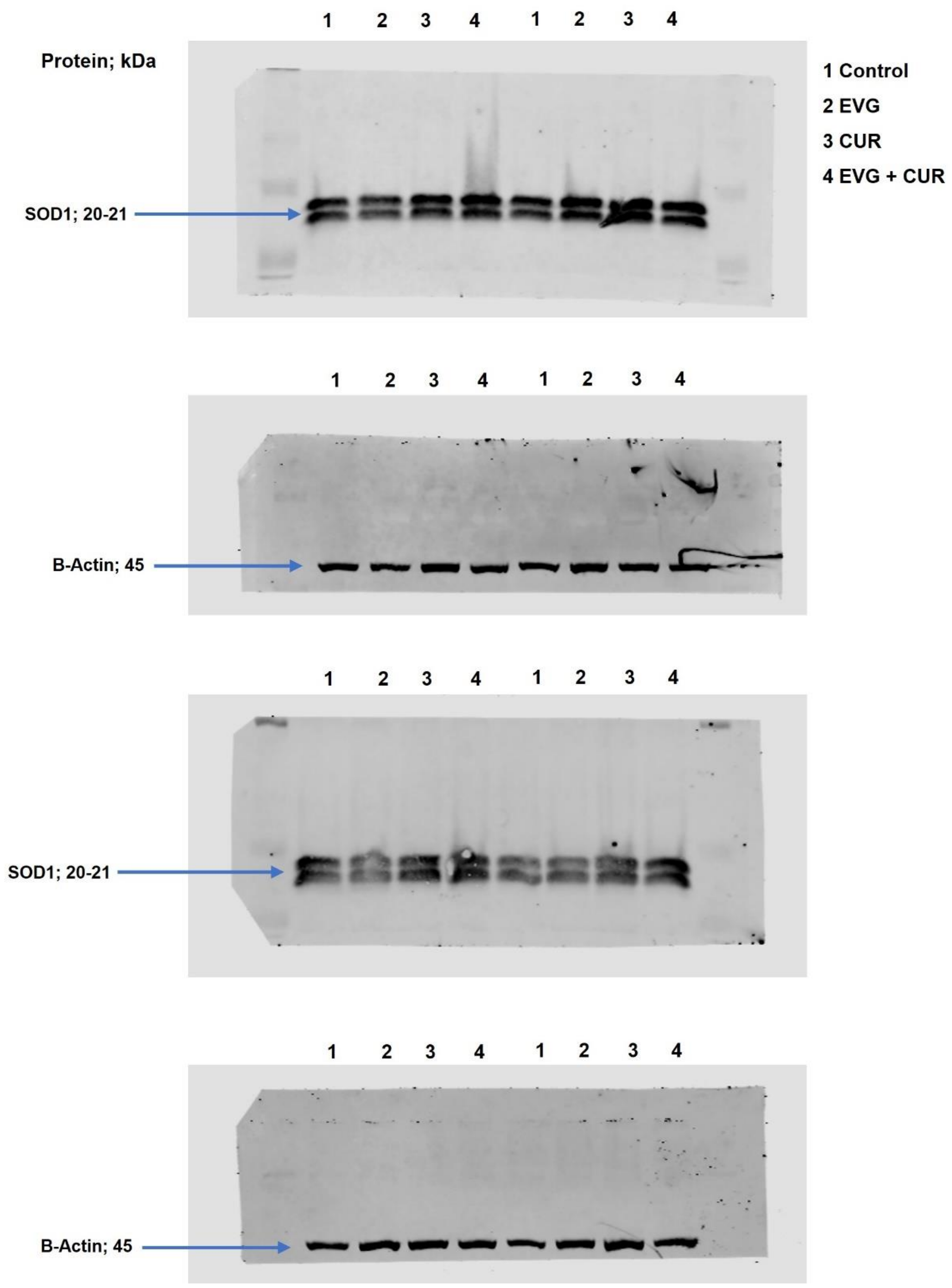

1 2 3 4 1 2 3 4
Protein; kDa
1 Control
2 EVG
3 CUR
4 EVG + CUR
SOD1; 20-21
1 2 3 4 1 2 3 4
B-Actin; 45
1 2 3 4 1 2 3 4
SOD1; 20-21
1 2 3 4 1 2 3 4
B-Actin; 45

Supplementary Figure S3: Figure 6 original blots b & c

The expression of IL-1β, TNF-α proteins were measured in U1 macrophages ($n = 4$) by Western blot.

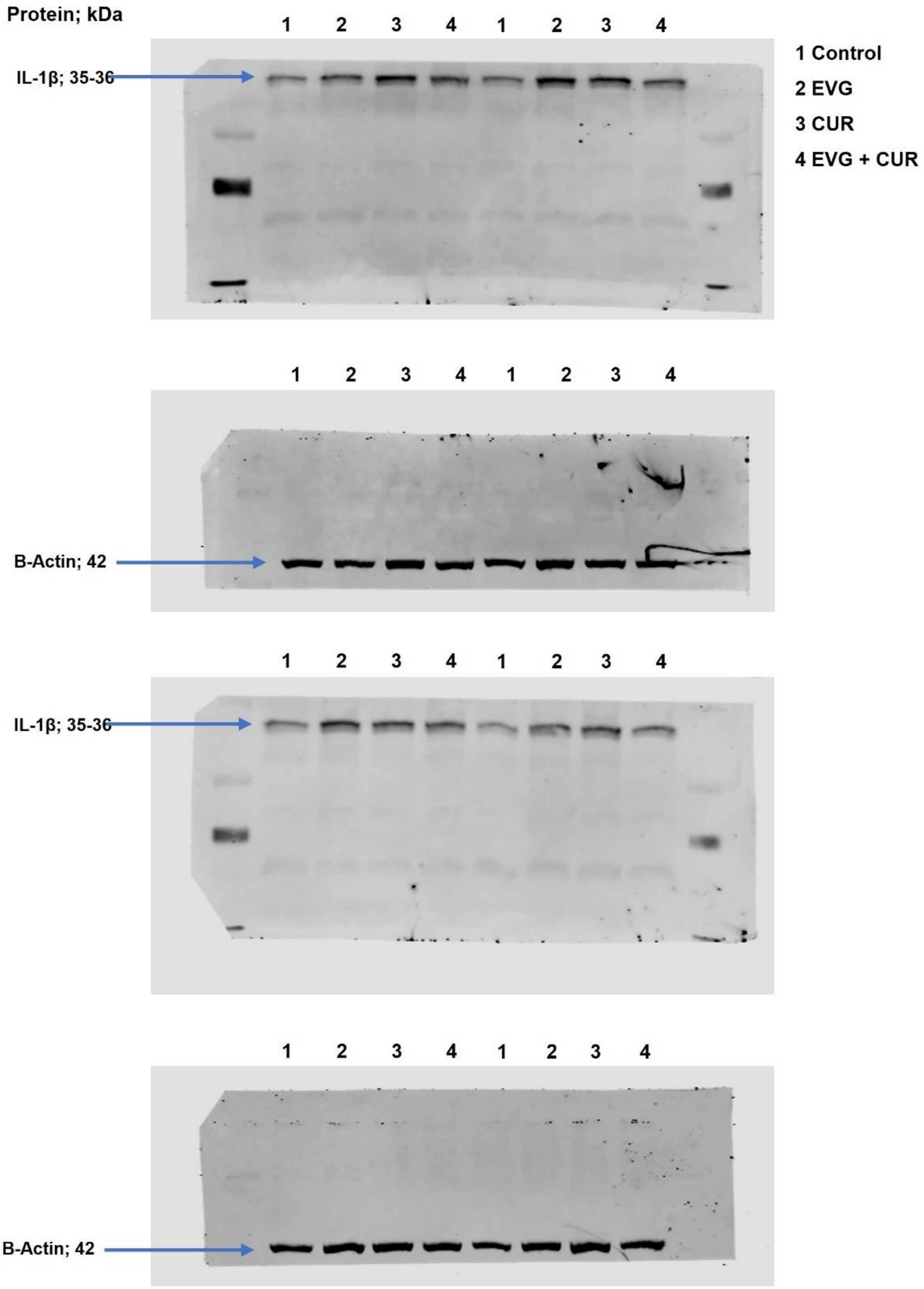

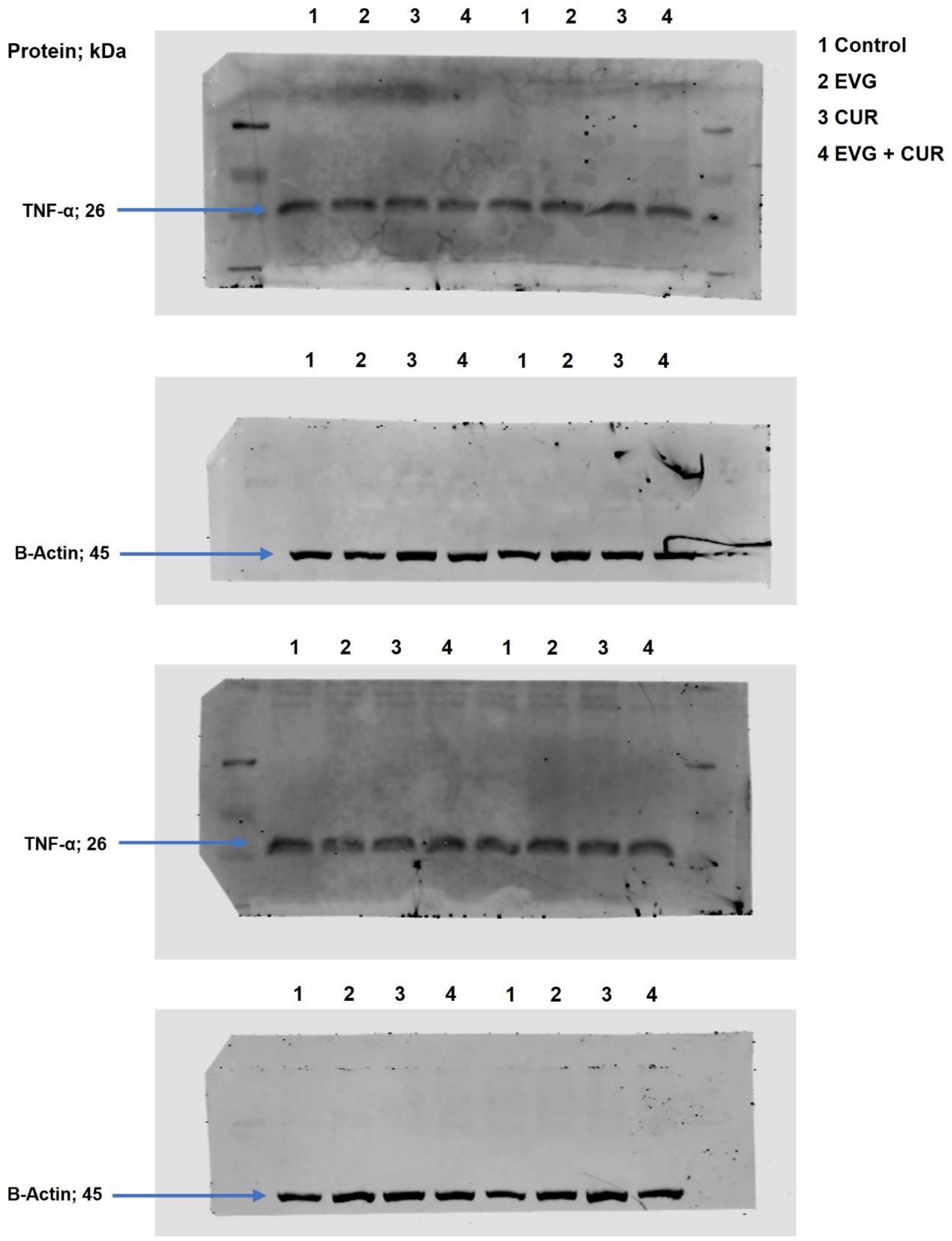
Protein; kDa
1 2 3 4 1 2 3 4
1 Control
2 EVG
3 CUR
4 EVG + CUR
TNF-α; 26
1 2 3 4 1 2 3 4
B-Actin; 45
1 2 3 4 1 2 3 4
TNF-α; 26
1 2 3 4 1 2 3 4
B-Actin; 45
